# IP-10, p53, and Foxp3 Expression in Hepatocytes of Chronic Hepatitis B Patients with Cirrhosis and Hepatocellular Carcinoma

**DOI:** 10.5005/jp-journals-10018-1188

**Published:** 2016-12-01

**Authors:** Umme Shahera, Saifullah Munshi, Munira Jahan, Afzalun Nessa, Shahinul Alam, Shahina Tabassum

**Affiliations:** 1 Department of Virology, Bangabandhu Sheikh Mujib Medical University, Shahabag, Dhaka, Bangladesh; 2 Department of Hepatology Bangabandhu Sheikh Mujib Medical University, Shahabag, Dhaka, Bangladesh

**Keywords:** Cirrhosis, Gene expression, Hepatitis B virus, Hepatocellular carcinoma.

## Abstract

**Aim:**

Elucidating differences in gene expression may be useful in understanding the molecular pathogenesis and for developing specific markers for the outcome of hepatitis B virus (HBV) infection. In the present study, expressions of host gene interferon gamma-inducible protein (IP-10), p53, and Foxp3 were studied in hepatocytes of patients with chronic HBV infection to determine a possible link between selected host gene expression and the outcome of HBV infection.

**Materials and methods:**

The study was conducted in 60 patients with chronic HBV infection and they were divided into four groups: HBV-positive cirrhosis (n = 15), HBV-negative cirrhosis (n = 15), HBV-positive hepatocellular carcinoma (HCC) (n = 15) and HBV-negative HCC (n = 15). Total messenger ribonucleic acid (mRNA) extraction was done followed by complementary deoxyribonucleic acid (cDNA) synthesis, and finally gene expression was performed using real-time polymerase chain reaction (PCR) technique.

**Results:**

IP-10 and p53 gene expressions were lower in HBV-positive cirrhosis, and Foxp3 gene expression was upregulated in HBV-positive cirrhosis in comparison to HBV-negative cirrhosis. The expressions of all the three genes were upregulated among HBV-positive HCC in comparison to HBV-negative HCC. The expression of IP-10, p53, and Foxp3 genes was upregulated in HBV-positive HCC in comparison to HBV-positive cirrhosis.

**Conclusion:**

This study indicates that there are variations in the expression of the selected genes among cirrhosis and HCC patients with or without HBV. All the three selected genes were more or less upregulated in HBV-positive HCC patients, but only Foxp3 expression was upregulated in HBV-positive cirrhosis. These three particular genes may have a role in the molecular pathogenesis and clinical outcome of HBV-positive cirrhosis and HCC patients. These aspects need further evaluation by studies with larger numbers of cirrhosis and HCC patients.

**How to cite this article:**

Shahera U, Munshi S, Jahan M, Nessa A, Alam S, Tabassum S. IP-10, p53, and Foxp3 Expression in Hepatocytes of Chronic Hepatitis B Patients with Cirrhosis and Hepatocellular Carcinoma. Euroasian J Hepato-Gastroenterol 2016;6(2):149-153.

## INTRODUCTION

Hepatitis B virus (HBV) is a noncytopathic, hepatotropic deoxyribonucleic acid (DNA) virus which is capable of inducing acute and chronic necro-inflammatory liver injury.^1^ More than 350 million people worldwide are chronically infected with HBV, causing acute and chronic necro-inflammatory liver injury and promoting hepatocarcinogenesis.^2^ In fact, chronic HBV infection causes liver cirrhosis (LC) leading to hepatocellular carcinoma (HCC), estimated over 50% of all HCC cases worldwide.^3^ Every year about one million people die of HBV-related cirrhosis or HCC.^4^

A strong genetic component with modified gene expression seems to be the major driving force affecting the course of viral hepatitis.^5^ Differential host gene regulation contributes to the different outcomes of HBV infection. However, both virological and host immunological factors also play important roles in determining the outcome.^6^ Genes, such as interferon gamma-inducible protein (IP-10), p53, and Foxp3 have been previously reported to have significant fold changes in different complementary DNA microarray analysis of genes expressed in hepatocytes in patients with HCC.^7,8^

Interferon gamma-inducible protein-10 is a chemokine produced by endothelial cells, activated T cells, and hepatocytes, exerting its effects mainly through a G protein-coupled receptor CXCR3.^9^ Chemokine IP-10 may play an important role in trafficking inflammatory cells to the local focus in the liver and induce the development of the chronicity of hepatitis B. Excessive inflammation of local liver tissues in chronic hepatitis B may eventually lead to LC.^7^

On the contrary, the development of HCC is a multistage process. Several extrinsic factors, such as aflatoxin, HBV, nutrition, alcohol, and trace elements, are thought to initiate and/or promote hepatocarcinogenesis. Alteration of p53 status is an important intrinsic factor in this process as p53 is essential for preventing inappropriate cell proliferation and maintaining genome integrity following genotoxic stress.^10^ Inactivation of wild-type (wt) p53 by point mutation, allelic deletion, or complex formation with cellular or viral protein is a common and crucial event in the occurrence of progression of liver cancer. The status of p53 is therefore crucial to the response of HCC to some therapies.^11^

Foxp3 T_reg_ can inhibit activation, proliferation, and effector functions of several kinds of other immune cells, including CD4+ and CD8+ T cells, natural killer (NK) and NKT cells, and dendritic cells. As such, Foxp3 T_reg_ cells are widely considered to be the principal mediator of dominant self-tolerance immune homeostasis.^8^ In HBV infection, Foxp3 T_reg_ cells have been associated with the incidence and extent of liver damage.^12^ A specific molecular marker of regulatory T cells, Foxp3, exerts great influence on the development and function of T_reg_ cells. The increased levels of T_reg_ cells are also associated with increased HBV–DNA levels, and depletion of T_reg_ cells results in increased HBV-specific CD4+ and CD8+ T-cell proliferation and interferon (IFN) production.

## MATERIALS AND METHODS

After obtaining informed written consent, 60 patients with chronic HBV infection were selected for this study. Ultrasound-guided fine needle aspiration cytology was performed at the Department of Hepatology of Bangabandhu Sheikh Mujib Medical University (BSMMU) by trained hepatologists. The samples were collected in RB (ribonucleic acid [RNA]-binding) buffer solution for extraction of RNA (Geneaid, Taipei, Taiwan). All the samples were transported under appropriate conditions to the Molecular Laboratory of the Department of Virology, BSMMU, for further procedure.

### RNA Extraction and Real-time polymerase chain reaction

Total RNA was extracted using Total RNA mini kit (tissue) (Geneaid, Taipei, Taiwan, catalog no-RT100) according to the manufacturer’s instructions. Complementary deoxyribonucleic acid (cDNA) was synthesized using cDNA synthesis Kit (Solis-biodyne, Tartu, Estonia); 5 µL of cDNA was used for polymerase chain reaction (PCR). The selected gene expression was analyzed by Step One PCR machine (Applied Biosystem, USA) using HOT FIRE Pol Evagreen qPCR Mix plus (Rox) (Solis-Biodyne, Tartu, Estonia). Glyceraldehyde 3-phosphate dehydrogenase (GAPDH) was used as internal control to normalize the PCR reaction of selected genes. The following primers were used (5′–3′) Foxp3 sense: CACAA-CATGCGACCCCCTTTCACC; Foxp3 antisense: AGGTTGTGGCGGATGGCGTTCTTC. p53 sense: CACGCTTCCCTGGATTGG; p53 antisense: TCAACCCACAGCTGCACA; IP-10 sense: GCCTCT-CCCATCACTTCCCTAC; IP-10 antisense: GAAGCAG-GGTCAGAACATCCAC. GAPDH sense: ATCCCATCACCATCTTCCAG; GAPDH antisense: ATGAGTCCTTCCACGATACC. Polymerase chain reaction was performed with initial denaturation at 95°C for 15 min, denaturation at 95°C for 15 seconds, annealing at 55°C for GAPDH, 53°C for IP-10, 58°C for p53 and 60°Cc for Foxp3 for 30 seconds, and lastly, extension at 72°C for 30 seconds. Fluorescence signals were measured after 40 PCR cycles.

The C_T_ values provided from the real-time PCR runs were imported into a spreadsheet program of Microsoft Excel. Duplicate samples from wells were collected at each time point and the mean was calculated. The data were analyzed using ΔΔC_T_ = (C_T_, Target – C_T_, GAPDH)_Target group_ – (C_T_, Target – C_T_, GAPDH)_Control group_. The fold change in the target gene, normalized to GAPDH and relative to the expression at time zero, was calculated for each sample using this equation.

### Statistical Analysis

Comparisons between HBV-positive cirrhosis and HCC and HBV-positive HCC and HBV-negative HCC were performed using Wilcoxon rank-sum (Mann–Whitney) test. Two-sample *t*-test was performed between HBV-positive cirrhosis and HBV-negative cirrhosis. All statistical calculations were performed with Statistical Package for the Social Sciences (SPSS) version 16.0. Differences were considered statistically significant when the p-value was <0.05 and highly significant when p-value was <0.001.

## RESULTS

Comparison between HBV-positive HCC and HBV-negative HCC showed that IP-10 (p < 0.001), p53 (p < 0.05), and Foxp3 (p < 0.001) were significantly increased in HBV-positive HCC (Table 1). The upregulation of IP-10, p53, and Foxp3 expression was 19.32-, 2.61-, and 14.48-fold in HBV-positive HCC patients when compared with HBV-negative HCC patients (1.00-, 1.00-, and 1.00-fold; not shown).

**Table 1:** **Table 1:** Relative expression of the examined genes among HBV-positive HCC and HBV-negative HCC. Two-sample Wilcoxon rank-sum (Mann–Whitney) test

*Gene*		*Groups*		*Sample no*		*Ranksum*		*Expected*		*p-value*	
IP-10		HBV-positive HCC		15		345		232.5		< 0.001	
		HBV-negative HCC		15		120		232.5			
p53		HBV-positive HCC		15		292.5		232.5		< 0.05	
		HBV-negative HCC		15		172.5		232.5			
Foxp3		HBV-positive HCC		15		345		232.5		< 0.001	
		HBV-negative HCC		15		120		232.5			

Interferon gamma-inducible protein-10, p53, and Foxp3 were all significantly increased (p < 0.001) in HBV-positive HCC patients than in HBV-positive cirrhosis patients when compared between these two groups (Table 2). The up regulation of IP-10, p53, and Foxp3 expression was 6.5-, 2.56-, and 6.32-fold respectively, in HBV-positive HCC when compared with HBV-positive cirrhosis, where these were 1.00-, 1.00-, and 1.00-fold respectively (not shown).

**Table 2:** **Table 2:** Relative expression of the studied genes among HBV-positive HCC and cirrhosis. Two-sample Wilcoxon rank-sum (Mann–Whitney) test

*Gene*		*Groups*		*Sample no*		*Ranksum*		*Expected*		*p-value*	
IP-10		HBV-positive HCC		15		345		232.5		< 0.001	
		HBV-positive cirrhosis		15		120		232.5			
p53		HBV-positive HCC		15		336		232.5		< 0.001	
		HBV-positive cirrhosis		15		129		232.5			
Foxp3		HBV-positive HCC		15		336		232.5		< 0.001	
		HBV-positive cirrhosis		15		129		232.5			

**Graph 1:**
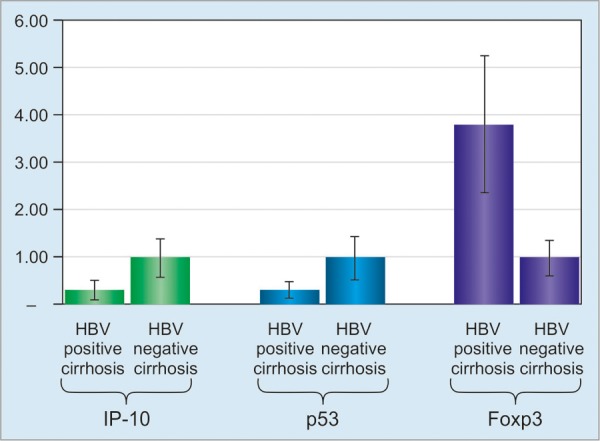
Comparison of IP-10, p53, and Foxp3 expression among HBV-positive cirrhosis and HBV-negative cirrhosis

However, comparison between HBV-positive cirrhosis and HBV-negative cirrhosis showed that IP-10 (p = 0.003) and p53 (p = 0.0072) were significantly decreased in HBV-positive cirrhosis, but Foxp3 (p = 0.0004) was significantly increased in HBV-positive cirrhosis (Graph 1). The downregulation of IP-10 and p53 was 0.319- and 0.330-fold respectively, in HBV-positive cirrhosis when compared with HBV-negative cirrhosis, which were 1.00- and 1.00-fold respectively (Graph 1). The upregulation of Foxp3 gene expression was 3.81-fold in HBV-positive cirrhosis when compared with HBV-negative cirrhosis, which was 1.00-fold (Graph 1).

## DISCUSSION

To understand gene dysregulation in liver diseases, the cDNA microarray technology has been used for global gene expression analysis of hepatitis viruses,^13^ chronic viral hepatitis,^14^ cirrhotic nodule,^15^ dysplastic nodules,^16^ and HCC.^17^ Although a few studies have investigated dysregulated genes in cirrhotic nodules, very little is known about the genes implicated in the pathophysiologic change of LC, or their relationship with the degree of decompensation. Liver cirrhosis is anatomically defined as diffuse fibrosis and regenerating nodules of liver, and patients die from various complications associated with cirrhotic decompensation or HCC, with an annual incidence of between 2.5 and 7% of cirrhotic liver cases.^18^ In this study, IP-10, p53, and Foxp3 genes were selected to elucidate differences in gene expression, which may be useful to understand the molecular pathogenesis and develop specific markers at different stages of viral hepatitis B, especially cirrhosis and HCC.

In the present study, IP-10 gene was significantly higher in HBV-positive patients with liver cancer than HBV-positive cirrhosis. Similarly, the expression of IP-10 was significantly higher in HBV-positive HCC than HBV-negative HCC patients. However, the expression of IP-10 gene was reduced in HBV-positive cirrhosis in comparison with HBV-negative cirrhosis. The higher expression of IP-10 in patients with hepatitis B may play a decisive role in the recruitment and accumulation of monocytes and lymphocytes within the liver tissue through interaction with its CXCR3 receptors expressed in target cells.^8^ These data suggest that IP-10 is elevated in HBV-infected liver tissue and may play an important role in HBV infection-induced hepatitis, especially more in HCC.

Chronic HBV infection is the major risk factor for the development of HCC through a multistep pathway that involves viral- and nonviral-dependent pathophysiological steps. Hepatic expression of the nuclear proliferative marker p53 oncoprotein was found to be associated with a poor outcome. Previously, the expressions of the proliferative marker p53 have been evaluated in HBV-related HCC. p53 was highly expressed and significantly related to hepatocarcinogenesis. p53 markers were found to be significantly higher in advanced stages, portal invasion, and intrahepatic metastasis and were associated with poor outcome.

The present study determined that p53 was elevated in HBV-infected liver tissue of HCC patients than cirrhosis with HBV, and the difference was statistically significant. The upregulation of p53 gene between HBV-positive HCC and HBV-negative HCC was also statistically significant. These findings suggest that p53 gene overexpression closely correlates with tumor progression in HCC.

A specific molecular marker of regulatory T cells, Foxp3, exerts great influence on the development and function of regulatory T_reg_ cells. The increased levels of regulatory T_reg_ cells are also associated with increased HBV–DNA levels, and depletion of regulatory T_reg_ cells results in increased HBV-specific CD4+ and CD8+ T-cell proliferation and interferon production.

In the present study, FOXP3 gene expression was higher in HBV-positive HCC than HBV-negative HCC. The findings of higher number of T_reg_ cells in patients with HCC have implication both in the pathogenesis of tumor and in the design of immunotherapy against it. The elimination of CD+CD25+ T cells may enhance tumor immunity when combined with the current attempts to augment immunogenicity of tumor cells.^19^

Foxp3 regulates tumor progression by expressing not only in T_regs_, but also in tumor cells of HCC, and it may also be functional in tumor cells of HCC. Comparison of Foxp3 expression between HBV-positive cirrhosis and HBV-positive HCC in the present study observed that upregulation of Foxp3 was more in HBV-positive HCC patients, thus supporting that Foxp3 is associated with the incidence and extent of liver damage in HBV patients.^12^

## CONCLUSION

The present study concluded that there were variations in the expression of the selected genes among cirrhosis and HCC patients with or without HBV. All the three selected genes were more or less upregulated in HBV-positive HCC patients, but only Foxp3 expression was upregulated in HBV-positive cirrhosis. These three particular genes may be responsible for the molecular pathogenesis and clinical outcome of HBV-positive cirrhosis and HCC patients.

## CLINICAL SIGNIFICANCE

Thus, the IP-10, p53, and Foxp3 genes may be used as a marker for HCC. However, these aspects need to be evaluated further by studies among larger numbers of cirrhosis and HCC patients.
